# Reduced apparent fiber density in the white matter of premature-born adults

**DOI:** 10.1038/s41598-020-73717-6

**Published:** 2020-10-14

**Authors:** Aurore Menegaux, Dennis M. Hedderich, Josef G. Bäuml, Andrei Manoliu, Marcel Daamen, Ronja C. Berg, Christine Preibisch, Claus Zimmer, Henning Boecker, Peter Bartmann, Dieter Wolke, Christian Sorg, Philipp Stämpfli

**Affiliations:** 1 Department of Diagnostic and Interventional Neuroradiology, School of Medicine, Technical University of Munich, Munich, Germany; 2 TUM Neuroimaging Center, School of Medicine, Technical University of Munich, Munich, Germany; 3 Department of Psychiatry, Psychotherapy and Psychosomatics, Psychiatric Hospital, University of Zurich, Zurich, Switzerland; 4 Wellcome Centre for Human Neuroimaging, University College London, London, UK; 5 Centre for Computational Psychiatry and Ageing Research, Max Planck University College London, London, UK; 6 Functional Neuroimaging Group, Department of Radiology, University Hospital Bonn, Bonn, Germany; 7 Department of Neonatology, University Hospital Bonn, Bonn, Germany; 8 Department of Psychology, University of Warwick, Coventry, UK; 9 Warwick Medical School, University of Warwick, Coventry, UK; 10 Department of Psychiatry, School of Medicine, Technical University of Munich, Munich, Germany; 11 MR-Center of the Department of Psychiatry, Psychotherapy, and Psychosomatics and the Department of Child and Adolescent Psychiatry, Psychiatric Hospital of the University of Zurich, University of Zurich, Zurich, Switzerland

**Keywords:** Development of the nervous system, Neural circuits, Brain, Neonatal brain damage, White matter injury

## Abstract

Premature-born adults exhibit lasting white matter alterations as demonstrated by widespread reduction in fractional anisotropy (FA) based on diffusion-weighted imaging (DWI). FA reduction, however, is non-specific for microscopic underpinnings such as aberrant myelination or fiber density (FD). Using recent advances in DWI, we tested the hypothesis of reduced FD in premature-born adults and investigated its link with the degree of prematurity and cognition. 73 premature- and 89 mature-born adults aged 25–27 years underwent single-shell DWI, from which a FD measure was derived using convex optimization modeling for microstructure informed tractography (COMMIT). Premature-born adults exhibited lower FD in numerous tracts including the corpus callosum and corona radiata compared to mature-born adults. These FD alterations were associated with both the degree of prematurity, as assessed via gestational age and birth weight, as well as with reduced cognition as measured by full-scale IQ. Finally, lower FD overlapped with lower FA, suggesting lower FD underlie unspecific FA reductions. Results provide evidence that premature birth leads to lower FD in adulthood which links with lower full-scale IQ. Data suggest that lower FD partly underpins FA reductions of premature birth but that other processes such as hypomyelination might also take place.

## Introduction

Premature birth, as defined by birth before 37 weeks of gestation or with birth weight below 2500 g is associated with long term impairments in brain structure, particularly in white matter (WM)^1–3^.

At the microscopic level, histological studies of animal models of prematurity and neuropathological studies in non-surviving premature-born infants reveal neuronal and axonal degeneration^4^ as well as aberrant maturation of oligodendrocytes progenitor cells (OPCs), pre-oligodendrocytes (pre-OLs), and associated myelination^5,6^. For example, initial insults such as hypoxia/ischemia, inflammation or infection lead to primary pre-OLs injury or death and thus—due to microglia activation and neuroinflammation—to dysmaturation, resulting in impaired myelination and axonal damage^1,4–11^. However, little is known about the course of WM changes in surviving children and adults due to both the lack of neuropathological assessments and the non-specificity of in-vivo imaging findings, mainly by diffusion tensor imaging (DTI).

DTI is the most common method used to map WM of the human brain in-vivo. It relies on diffusion weighted imaging (DWI) data on which a tensor model is applied to quantify the magnitude and directionality of water diffusion in brain tissue, thus allowing inferences about WM microstructure^12,13^. Several measures can be derived from the tensor model, particularly fractional anisotropy (FA), reflecting the degree of directionality of water diffusion^14^. Widespread reductions in FA have been reported in premature-born individuals, from infancy^15–19^ to adulthood^2,20–24^. These FA alterations have been shown to be associated with the degree of prematurity and to be relevant for cognitive abilities^2,20^. FA reductions are, however, not specific for underpinning microscopic features, such as axonal degeneration, a reduction in packing density (i.e., fiber density), demyelination or gliosis^25–31^. Moreover, as DTI cannot resolve multiple fiber orientations, FA measures have been shown to be inadequate in regions of complex fiber geometries such as crossing fibers or multiple fiber populations, thus rendering the interpretation of FA changes difficult^32–34^. Consequently, although DTI has proven altered WM microstructure in vivo, it is unspecific and not well suited to properly characterize microstructural changes particularly in individuals born prematurely.

Recent developments in DWI data acquisition and analysis, raise hope to overcome this non-specificity of FA by inferring more specific microstructural features of the neuronal tissue such as axonal diameter and apparent fiber density^35^. Several methods have been proposed which make use of imaging protocols acquiring a higher number of gradient directions and higher b-values, combined with data analyses approaches based on higher order and multi-compartment models^35–41^. Indeed, these techniques assume that the tissue is composed of several different compartments such as for example axons, glial cells and extra-axonal space and cerebrospinal fluid (CSF). Each compartment is characterized by a different pattern of water diffusion (restricted for intra-axonal, hindered for extra-axonal, isotropic for CSF) and their combination is employed to model the DWI signal measured in each voxel. The microstructure informed tractography model from Daducci and colleagues (Convex optimization modeling for microstructure informed tractography, COMMIT^42^) combines this multi-compartment approach for the estimation of local microstructure properties of the tissue with the advantages of fiber tracking. First, a large set of candidate fiber tracts (streamlines) is estimated using one of the classical fiber tracking algorithms. In the second step, an optimal weight is determined for each streamline to best fit the measured diffusion signal according to a biologically motivated multi-compartment model, namely the ball stick and zeppelin model from Panagiotaki and colleagues which accounts for intra-axonal, extra-axonal and CSF compartments, respectively^35^. While the terminology for describing the intra-axonal restricted compartment varies considerably depending on the model being used, it is already common in the literature to refer to it as apparent fiber density (FD)^43,44^. Although several algorithms can be used for fiber tracking, the constrained spherical deconvolution (CSD) method enables the detection of multiple fiber directions within a single voxel thus improving the performance of fiber tractography considerably^45^. As COMMIT makes use of the CSD approach, it can handle multiple fiber populations and be reliably used in voxels containing crossing fibers (which have been shown to be present in 60 to 90% of all WM fiber voxels^32^). Moreover, it has been successfully employed to detect subtle apparent fiber density reductions in patient groups such as patients with amyotrophic lateral sclerosis and schizophrenia^46,47^. We thus aimed at employing this method to investigate FD in premature-born adults.

Remarkably, using a similar method, Pannek and colleagues found that FD was reduced in premature-born infants at term equivalent age, compared to mature-born ones and that FD was significantly positively correlated with gestational age (GA)^48^. Based on this discovery and on the histological findings of axonal degeneration and aberrant pre-OL maturation in animal models of hypoxia–ischemia and neuropathological studies of premature-born infants, we hypothesized that FD would be reduced in premature-born adults compared to mature-born ones and that these alterations would be related to the degree of prematurity. In order to investigate these hypotheses, we employed the microstructure informed tractography model, from Daducci and colleagues^42^ to derive FD measures from diffusion weighted imaging for a cohort of 73 very premature-born adults (i.e., very preterm and/ or with very low birth weight adults, VP/VLBW) and 89 full-term (FT) born adults. Associations between FD and the degree of prematurity, as measured by GA and birth weight (BW), were explored via correlations analyses. Similarly, the association between FD and neonatal medical complications as measured by the OPTI score (optimality neonatal) was assessed.

Furthermore, although no study has investigated the link between FD and full-scale intelligence quotient (FS-IQ) yet, it has previously been shown that FA was significantly associated with FS-IQ^2,22,49^. Thus, we aimed to assess the functional relevance of FD alterations with general cognitive abilities as defined by FS-IQ. Finally, in order to investigate the potential dependence of FD on FA changes, we related FD alterations with FA.

## Results

### Sample characteristics

The demographic characteristics of the study sample are listed in Table 1. By design, VP/VLBW individuals had significantly lower GA, (p < 0.001), BW (p =  < 0.001) and a higher OPTI neonatal score (p < 0.001) than FT subjects. VP/VLBW subjects also had significantly lower FS-IQ scores (p < 0.001). However, the two groups did not differ regarding age at scan (p = 0.54), sex (p = 0.93), maternal age (p = 0.66) and socioeconomic status at birth (p = 0.59).

**Table 1:** Sample characteristics.

	VP/VLBW (n = 73)			FT (n = 89)			p value
	M	SD	Range	M	SD	Range	
Sex(male/female)	44/29			53/36			0.93
Age (years)	26.8	± 0.6	25.8–28.0	26.8	± 0.8	25.6–28.9	0.54
GA (weeks)	30.4	± 2.1	25–36	39.7	± 1.0	37–42	** < 0.001**
BW (g)	1305	± 311	630–2070	3372	± 468	2120–4670	** < 0.001**
OPTI neonatal	9.1	± 2.6	3–14	0.4	± 0.7	0–3	** < 0.001**
SES^a^ (a.u.)	19/33/21		1–3	28/41/20		1–3	0.59
Maternal age (years)	29.3	± 4.9	16–38	29.3	± 5.0	18–42	0.66
Full-scale IQ (a.u.)	93.9	± 11.8	64–125	102.9	± 12.4	77–130	** < 0.001**

Statistical comparisons: Sex, SES with χ^2^ statistics; age, FS-IQ with two sample t-tests; GA, BW, maternal age with Mann–Whitney-U tests.

*BW* birth weight, *FT* full-term, *GA* gestational age, *IQ* intelligence quotient, *M* mean, *maternal age* maternal age at birth, *OPTI* standardized optimality scoring system, *SD* standard deviation, *SES* socioeconomic status at birth, *VP/VLBW* very preterm and/or very low birthweight.

^a^1 = upper class, 2 = middle class, 3 = lower class.

### Widespread lower apparent fiber density in premature-born adults

**Figure 1:**
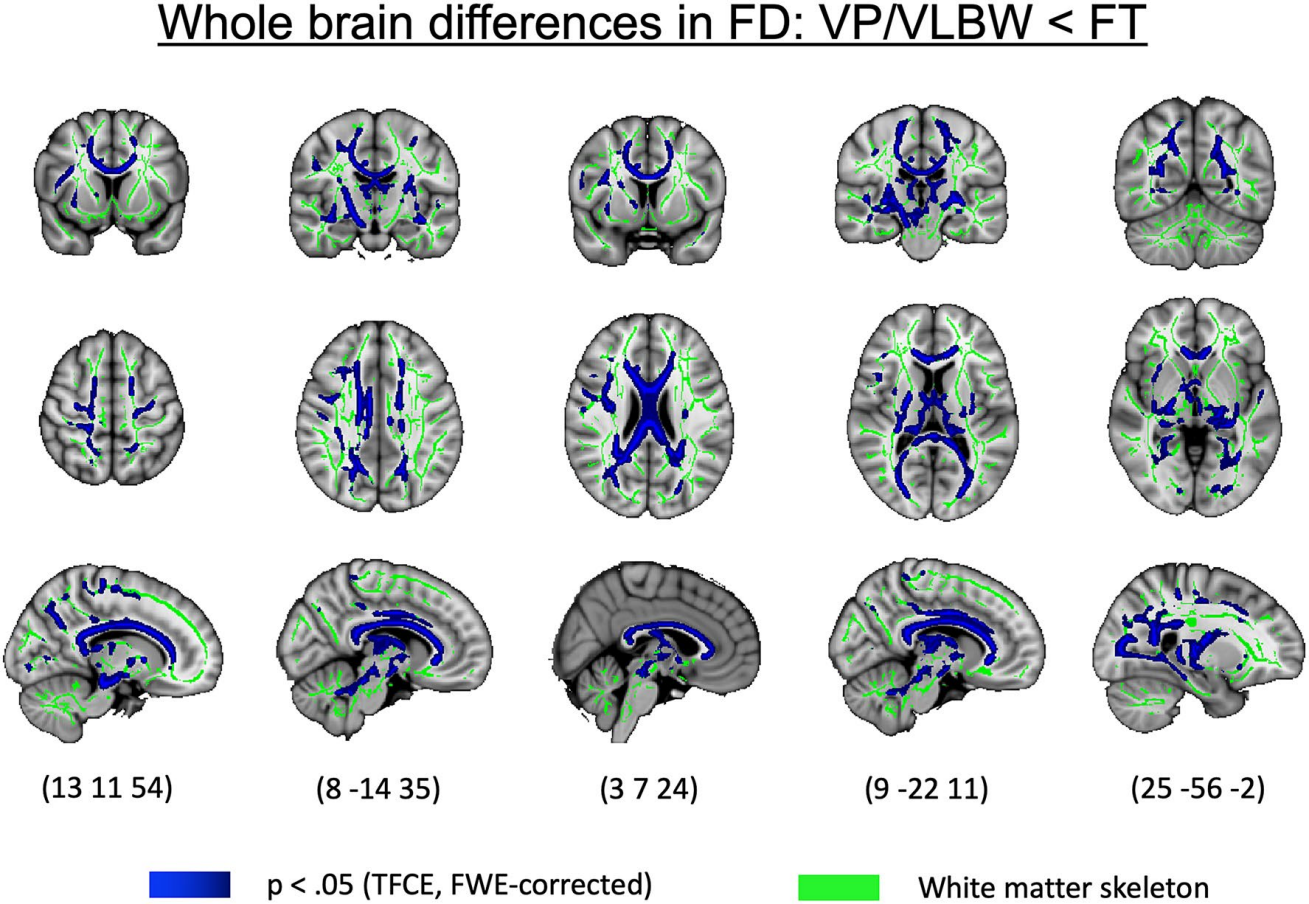
Whole brain group differences in FD. Coronal, axial, and sagittal views illustrating the group differences in FD between VP/VLBW adults and FT individuals overlaid on the T1-weighted brain image of the MNI152 structural standard template using FSLView Version 5.0.9 (https://fsl.fmrib.ox.ac.uk/fsl/fslwiki/FslView). Clusters of voxels with significantly lower FD in VP/VLBW adults (p < 0.05) are represented in blue on the green TBSS FA-based white matter skeleton. MNI coordinates are provided at the bottom.

In order to investigate group differences in FD between VP/VLBW and FT individuals, we performed a whole brain TBSS analysis controlling for sex and scanner. As shown in Fig. 1, VP/VLBW individuals exhibited significantly lower FD than FT subjects across 30,698 voxels (pFWE < 0.05, threshold-free cluster enhancement (TFCE) corrected). Most affected tracts included the genu, body and splenium of the corpus callosum, bilateral anterior, posterior and retrolenticular parts of the internal capsule, bilateral external capsule, corona radiata (anterior, superior and posterior part bilaterally), fornix, cerebral peduncle, sagittal stratum left and right (which included the longitudinal fasciculi and inferior-fronto-occipital fasciculi) as well as bilateral posterior thalamic radiations. Details on the number of voxels affected in each tract can be found in supplementary table S1. We did not find significantly higher FD in the VP/VLBW group than in the FT group.

**Figure 2:**
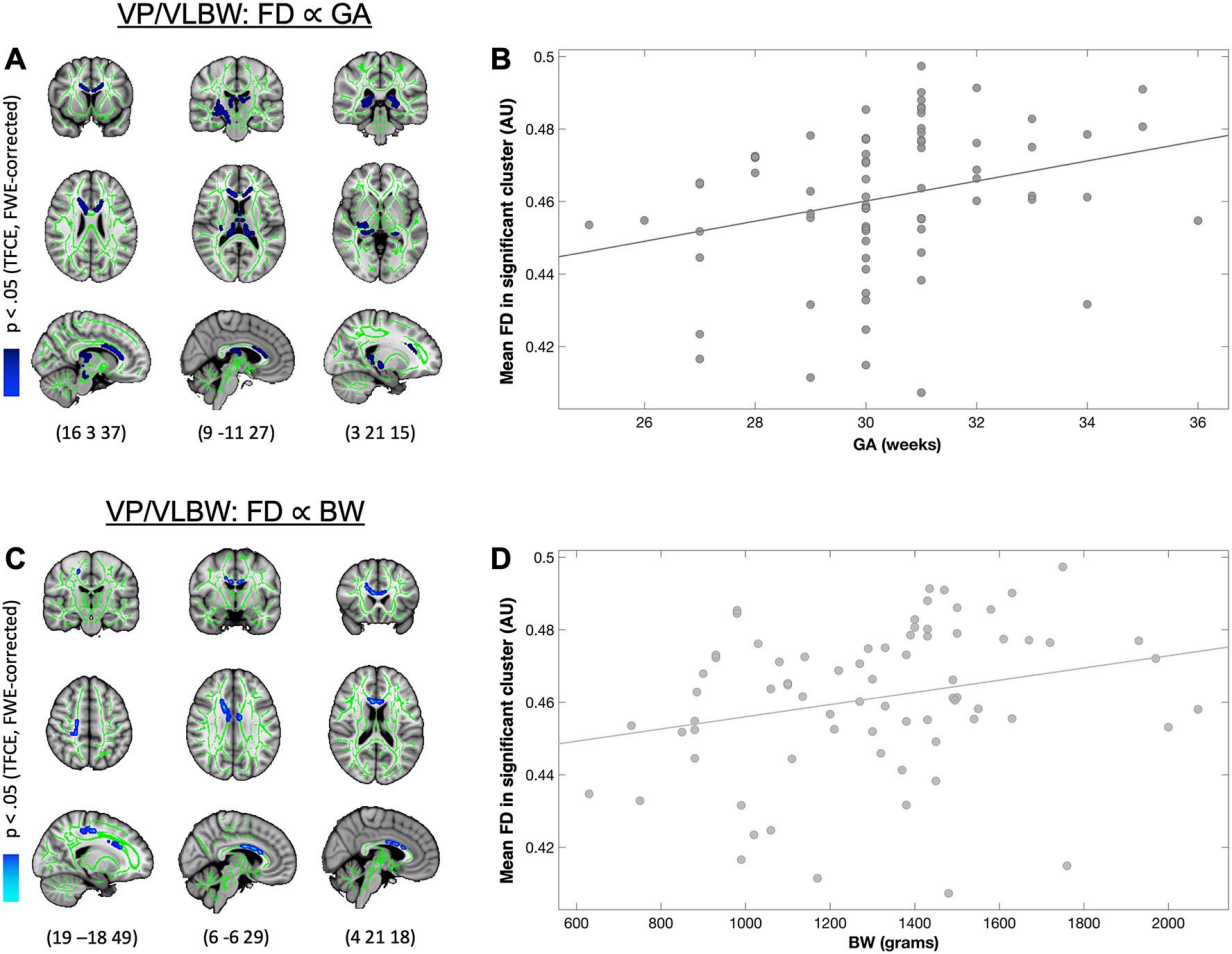
Association between lower FD and birth-related variables in VP/VLBW adults. A and B show the association between FD (from the clusters of voxels significantly different between groups in Fig. 1) and GA in the VP/VLBW group. In (**A**), coronal, axial, and sagittal views illustrate the voxelwise significant positive association between lower FD and GA in VP/VLBW adults. Dark blue represents the FD voxels significantly positively associated with GA (p < 0.05). (**B**) For visualization only, the association between mean lower FD and GA is shown. The FD values were averaged from all voxels where FD was significantly lower in VP/VLBW adults compared to FT individuals (Fig. 1). Similarly, C and D show the association between FD and BW in VP/VLBW adults. In (**C**) coronal, axial, and sagittal views illustrate the voxelwise significant positive association between lower FD and BW in VP/VLBW adults. Light blue represents the FD voxels significantly positively associated with BW (p < 0.05). (**D**) For visualization, the association between mean lower FD and BW is shown. FD values were averaged from all voxels where FD was significantly lower in VP/VLBW adults compared to FT individuals (Fig. 1). MNI coordinates are provided at the bottom. The results in A and C are presented on the T1-weighted brain image of the MNI152 structural standard template using FSLView Version 5.0.9 (https://fsl.fmrib.ox.ac.uk/fsl/fslwiki/FslView).

To further ensure that lower FD in premature-born adults was linked to the degree of prematurity, we performed correlation analyses between FD and birth-related variables GA, BW and OPTI, respectively, in the VP/VLBW group in the cluster of voxels that showed significant group differences in FD. We found a significant positive correlation between FD and GA (Fig. 2A,B) in 3477 voxels (p_FWE_ < 0.05, TFCE corrected) located mainly in the genu and body of the corpus callosum, the right cerebral peduncle, the right posterior and retrolenticular part of the internal capsule and right external capsule as well as in the fornix. We also found a significant positive correlation between FD and BW (Fig. 2C,D) in 700 voxels (p_FWE_ < 0.05, TFCE corrected) located in the right anterior corona radiata and the body and genu of the corpus callosum. Details on the number of significant voxels for each tract can be found in Table S2. Furthermore, we did not find any significant negative association between FD and GA or between FD and BW. Finally, no significant positive or negative association was found between FD and OPTI neonatal score.

### Altered apparent fiber density is associated with lower FS-IQ in premature-born adults

**Figure 3:**
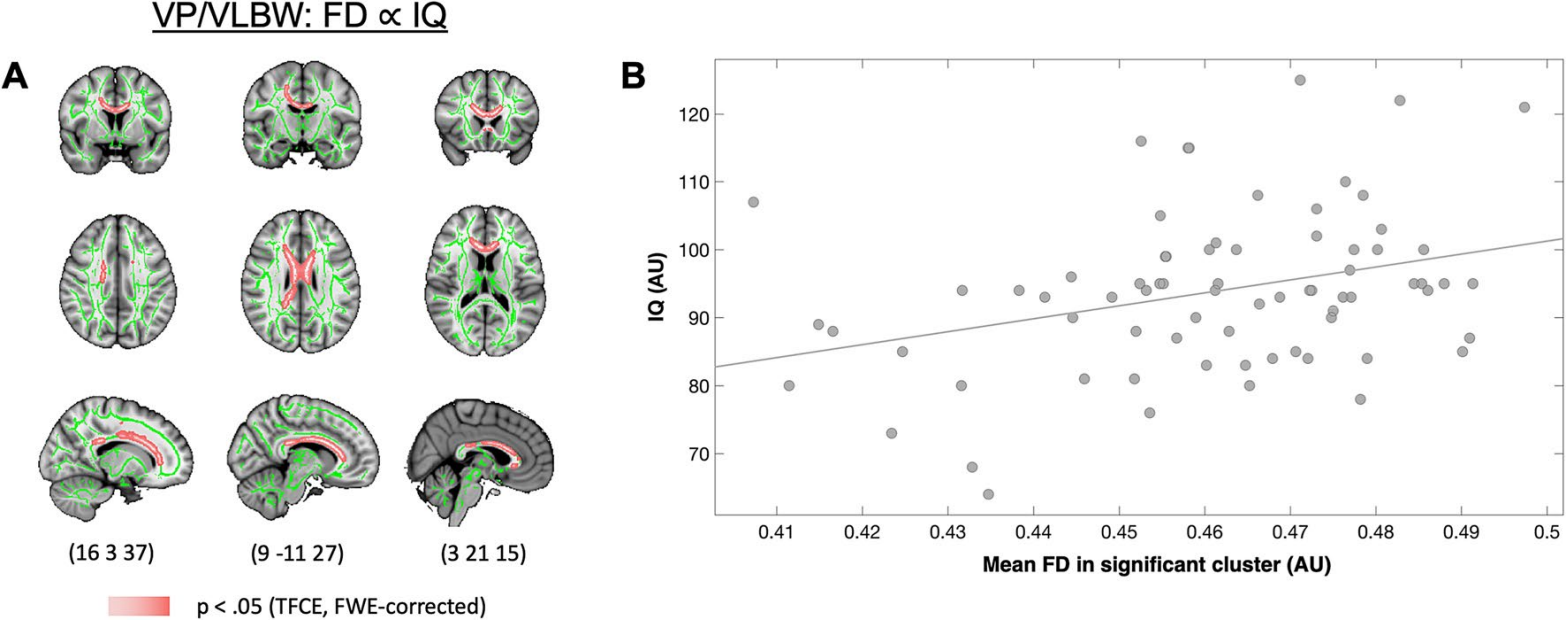
Association between lower FD and FS-IQ in VP/VLBW adults. A and B show the association between FD (from the clusters of voxels significantly different between groups) and FS-IQ in VP/VLBW adults. In (**A**) coronal, axial, and sagittal views illustrate the voxelwise significant positive association between lower FD and FS-IQ in VP/VLBW adults. Pink represents the FD voxels significantly positively associated with FS-IQ (p < 0.05). (**B**) In the upper right panel, for visualization, the association between mean lower FD and FS-IQ is shown. FD values were averaged from all voxels where FD was significantly lower in VP/VLBW adults compared to FT individuals (Fig. 1). MNI coordinates are provided at the bottom. The results in A are presented on the T1-weighted brain image of the MNI152 structural standard template using FSLView Version 5.0.9 (https://fsl.fmrib.ox.ac.uk/fsl/fslwiki/FslView).

In order to test the functional relevance of FD changes for general cognitive abilities, we investigated the association between FD and full-scale IQ in the VP/VLBW group. We found that FD was significantly positively correlated with FS-IQ in 2765 voxels in the body, genu and splenium of the corpus callosum as well as in the corona radiata (p_FWE_ < 0.05, TFCE corrected; Fig. 3 and Table S3). We did not find any significant negative association between FD and FS-IQ.

### Lower apparent fiber density overlaps with lower FA in premature-born adults

In order to better understand our findings of lower FD in VP/VLBW adults in relation to typical FA changes, we compared our group differences in FD with FA changes. To this end, we first, performed a whole brain FA TBSS analysis controlling for sex and scanner. As shown in Supplementary Fig. S2, we found widespread lower FA in VP/VLBW individuals compared to FT subjects in 51,000 voxels (pFWE < 0.05, TFCE corrected) spread across the genu, body and splenium of the corpus callosum, the bilateral corona radiata (anterior, posterior and superior parts), the bilateral internal (anterior, posterior and retrolenticular parts) and external capsules, the bilateral posterior thalamic radiations, bilateral inferior and superior longitudinal and fronto-occipital fasciculi, cingulum and cerebellar peduncles (Table S1).

**Figure 4:**
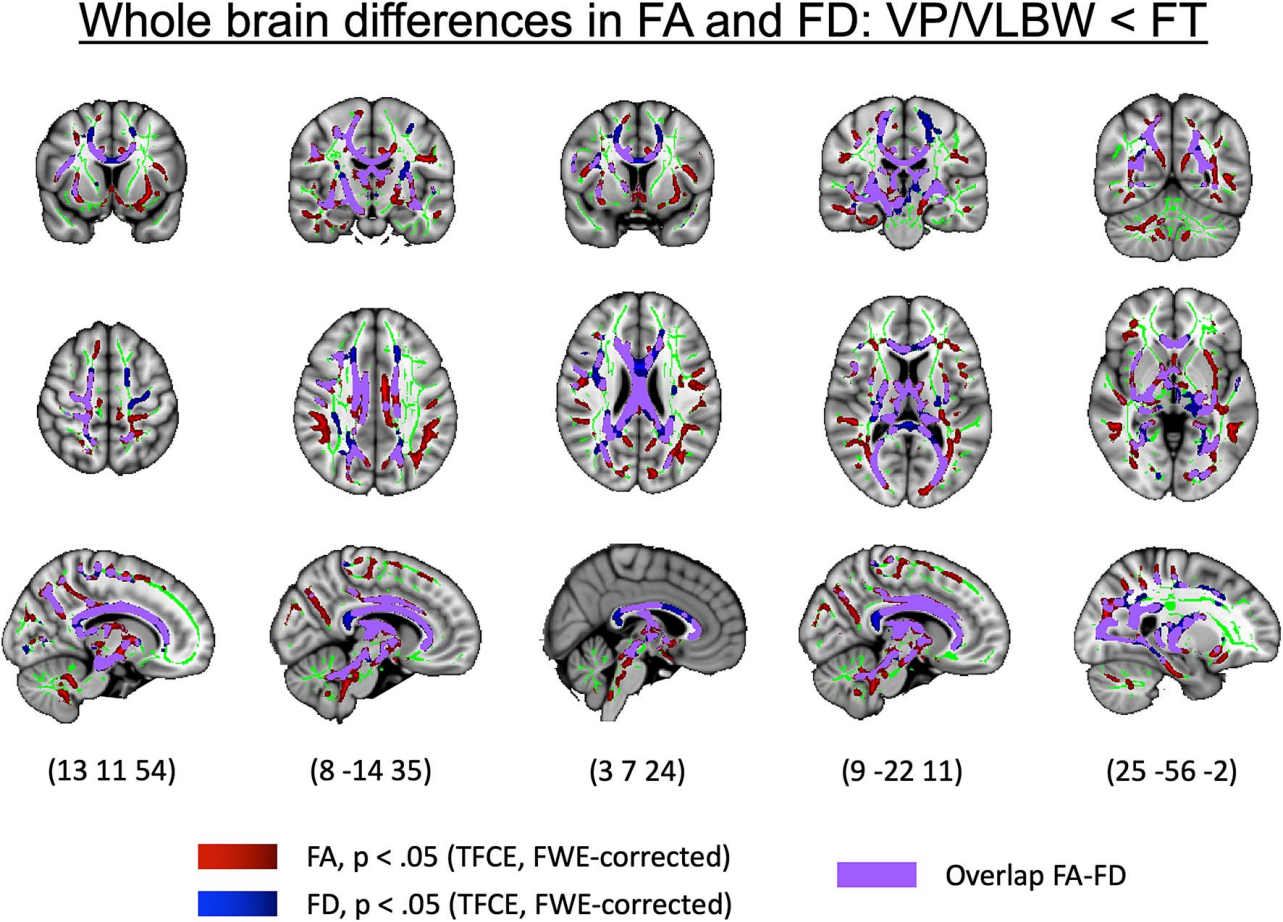
Overlap between lower FA and lower FD in VP/VLBW individuals. Coronal, axial, and sagittal views illustrating the overlap between lower FA and lower FD in VP/VLBW adults superimposed on the T1-weighted brain image of the MNI152 structural standard template using FSLView Version 5.0.9 (https://fsl.fmrib.ox.ac.uk/fsl/fslwiki/FslView). Clusters of voxels with significantly lower FA in VP/VLBW adults (p < 0.05) are represented in red, clusters with significantly lower FD in VP/VLBW adults (p < 0.05) are shown in blue and the voxels with overlap between significantly lower FD and FA in VP/VLBW adults (p < 0.05) are shown in purple on the green TBSS FA-based white matter skeleton. MNI coordinates are provided at the bottom.

Second, comparing FD and FA group difference maps, we found that 20,630 voxels out of the 30,698 voxels (67.2%) with lower FD also showed lower FA in VP/VLBW individuals compared to FT ones (Fig. 4). The overlap of lower FA and lower FD was primarily located in the body, genu and splenium part of the corpus callosum, the fornix, the right cerebral peduncle, bilateral internal (anterior, posterior and retrolenticular parts) and external capsules , bilateral corona radiata (anterior, posterior, superior parts), bilateral sagittal stratum and posterior thalamic radiations (Table S1).

## Discussion

Taking advantage of the recent advances in diffusion magnetic resonance imaging analyses to infer microstructural features of observed white matter changes, we investigated whether apparent fiber density (i.e. FD) was reduced in a group of 73 premature-born compared to 89 mature-born adults. We found that premature-born adults exhibited widespread lower FD compared to mature-born adults, and that lower FD in the corpus callosum, fornix, corona radiata and internal capsule were associated with the degree of prematurity as measured by gestational age and birth weight. Furthermore, we found that lower FD in the corpus callosum and corona radiata were associated with reduced general cognitive abilities as represented by the full-scale intelligent quotient (FS-IQ) score. Together, and to the best of our knowledge, our findings provide first-time evidence that premature birth leads to lower apparent fiber density in adulthood which seems to be linked with lower FS-IQ. Finally, lower FD largely overlapped with lower fractional anisotropy (FA), suggesting that lower FD might underlie lower FA. Our results also suggest that lower apparent fiber density might underpin lower FA after premature birth but that other processes such as hypomyelination might also take place.

In order to test our hypothesis of reduced apparent fiber density in premature-born individuals, we investigated voxel-wise group differences in FD obtained from the COMMIT method of Daducci and colleagues^42^ using TBSS. We found that FD was lower in premature-born individuals compared to mature-born ones. These alterations were widespread throughout the brain, particularly in association tracts such as the superior and inferior longitudinal fasciculi, the inferior and superior fronto-occipital fasciculi, and in commissural fibers including the body, genu and splenium of the corpus callosum. Findings of lower FD in such tracts are consistent with those from previous studies who reported microstructural alterations in similar tracts using DTI indices^20–22,49^. Although, to our knowledge, no study has investigated FD in VP/VLBW adults, two studies have previously reported lower FD in premature-born infants, children and early adolescents using fixel-based analysis^48,50^. Our results are in line with their findings of lower FD in the genu, body and splenium of the corpus callosum in premature-born infants^48^ and of lower FD in the corpus callosum, tapetum, inferior fronto-occipital fasciculus and fornix in premature-born children and adolescents^50^. Moreover, since more widespread lower FD was found in premature-born children than in infants, and in premature-born adolescents than in children, our report of lower FD in additional tracts in premature-born adults compared to adolescents as described by Kelly and colleagues is in line with these findings^50^.

Furthermore, we found that FD was significantly positively correlated with GA in the genu and body of the corpus callosum, the fornix, the bilateral corona radiata and the right posterior and retrolenticular part of the internal capsule of premature-born adults thus indicating the more premature, the lower the apparent fiber density. This finding is consistent with the one of a positive association between GA and FD previously reported in premature-born infants, children and adolescents^48,50,51^. We also detected a significant association between FD and BW (the lower the BW, the lower the FD). The significant association between FD and GA or BW was found for the most part in the commissural fibers which are among the first ones to mature and myelinate^52,53^. Thus, in line with previous findings^22^, our result of lower FD in premature-born adults together with previous report from lower FD in infants, children and adolescents^48,50^ would suggest that VP/VLBW impairs development of fibers very early on which is not compensated and persists until adulthood.

We did not, however, find any significant association between FD and OPTI score which is consistent with the reported lack of association between neonatal brain abnormality scores and axon density (represented by the intra-axonal volume compartment obtained via NODDI) by Kelly and colleagues^54^. We would speculate that neonatal medical complications might primarily affect other microstructural aspects of WM such as myelination while FD is preferentially impacted by the degree of prematurity (as measured by GA and BW). FD might also be affected by only one of the neonatal medical complications items that are included in the OPTI score such as the number of days on mechanical ventilation rather than by the majority which is why we did not find any significant association between FD and overall OPTI score.

Finally, lower FD in premature-born adults is in line with findings from histological studies of animal models of hypoxia–ischemia and neuropathological studies in premature-born non-surviving infants which reported neuronal and axonal degeneration^4^ as well aberrant maturation of OPCs, pre-OLs and myelination^5,6^. Interestingly, pre-OLs have been shown to have an important trophic role for axonal development and function^1^, similarly to oligodendrocytes^55^. Thus, pre-OLs dysmaturation likely impairs the maturation of axons^3^. Moreover, it has also been shown in mice that ablation of oligodendrocytes lead to secondary focal axonal changes and a reduction in axonal density^56–58^, which suggests that impaired maturation of oligodendrocytes can result in axonal damages and axonal degeneration. Thus, lower FD in VP/VLBW adults is consistent with those histological findings.

The importance of WM microstructure and particularly FA for general cognitive abilities has previously been reported in premature-born individuals, from infants to adults^2,18,22,49^. Nevertheless, given the previously mentioned lack of specificity of the FA measure, it remains unclear which component of WM specifically might be relevant for FS- IQ. We found a significant positive association between FS-IQ and FD in the genu, body and splenium of the corpus callosum as well as in the corona radiata in VPT/VLBW adults suggesting that general cognitive abilities increase with apparent fiber density in those tracts. Although, to our knowledge, no study has investigated the link between IQ and FD in premature-born adults, our finding is in line with the one from Kelly and colleagues who reported a positive association between IQ and axon density as represented by the intra-axonal volume compartment obtained from NODDI in several voxels including in the body of the corpus callosum in premature-born children^54^. Thus, our findings suggest that especially apparent fiber density might be relevant for FS-IQ. Further research is needed to confirm this finding.

In order to relate our findings of lower FD in VP/VLBW adults to previous findings, we also investigated FA changes. As previously reported^2^, we found widespread lower FA in VP/VLBW individuals compared to FT born ones. Most of the voxels with lower FD overlapped with voxels of lower FA in tracts previously reported to exhibit FA changes in premature-born individuals from infancy to adulthood^2,15–17,20–22^. This overlap of FD and FA changes, together with the successful use of FD to detect subtle WM alterations in ALS and schizophrenia^46,47^ would suggest that the FD measure obtained from the COMMIT model seems to be a reliable marker of WM microstructure. Indeed, unlike FA which has been shown to be inadequate in regions of complex fiber geometries^32,33^, FD profits from improved tractography algorithms and recent advances of higher order diffusion models^41,45,59^. It is thus a reliable measure in voxels containing crossing fibers^60^. Finally, the fact that most voxels with lower FD in VP/VLBW individuals were found in tracts which also exhibited lower FA suggests that low FA in VP/VLBW individuals is at least in part due to a reduction in apparent fiber density which is consistent with histological findings as described above. Similarly, our finding of an association of higher FD with higher FS-IQ score in some of the tracts where higher FA had been previously shown to be associated with better FS-IQ (genu, body and splenium of the corpus callosum) indicated that this relation might, at least in part, be driven by apparent fiber density.

Although we found most of lower FD in tracts exhibiting lower FA, some areas showed lower FA without lower FD suggesting that those differences might be due to other factors. One plausible explanation might be differences between premature-born and mature-born individuals regarding the angle or proportion of crossing fibers in those voxels. Given that FA measures have been shown to be inadequate in regions of complex fiber geometries such as crossing fibers or multiple fiber populations^32–34^, our finding might illustrate this issue and thus suggest that there are actually no true alterations of WM microstructure in these tracts but rather a difference in the geometry or proportion of crossing fibers. Another possible explanation for the finding of lower FA without lower FD might be that lower FA in these tracts could reflect other processes such as hypomyelination. Indeed, animal models of prematurity and neuropathological studies in non-surviving premature-born infants reveal aberrant maturation of OPCs, pre-OLs and myelination^5,6^. Interestingly, recent brain imaging studies using myelin-sensitive sequences suggest reduced myelin content in the WM of premature-born children, for example using magnetization transfer MRI^61^. Interestingly, using quantitative T1 imaging, Travis and colleagues did not only find reduced R1 (= 1/T1) in parts of the corpus callosum of premature-born children which was suggested to reflect differences in myelin content, but they also found that reduced R1 overlapped with lower FA in some but not all parts of the corpus callosum. Such results suggested that myelin content may drive some but not all the differences in WM between premature- and mature-born children and that other factors most likely contribute to FA differences^62^. In line with those findings, our study suggests that one of these factors might be apparent fiber density.

Our study has several limitations that should be considered with care. First, regarding the characteristics of our sample: Individuals with more severe impairments or multiple complications in the initial BLS cohort were more likely to be excluded in the initial screening for MRI. Thus there is a bias in our study towards VP/VLBW adults with higher FS-IQ and less severe neonatal complications. Accordingly, we assume that the findings of lower FD in VP/VLBW adults compared to FT born ones described here are rather conservative estimates of the differences that might be observed in the complete BLS sample. However, the sample used in this study was of large size (162 subjects) and of similar age thus enhancing the generalizability and robustness of our results. Second, the data were acquired in four different scanners which can influence apparent fiber density estimates. Although it cannot be certain that scanner type does not affect our findings, we have taken several steps to ensure it has a minimal effect such as keeping the DWI sequence parameters identical across scanners, comparing the signal-to-noise ratio which was not significantly different between scanners (one-way ANOVA with factor "scanner-ID" [Bonn 1, Bonn 2, Munich]; p = 0.81^63^) and finally, we used scanner type as a covariate of no interest in our statistical analyses. Thirdly, our diffusion weighted data were acquired with 32 diffusion directions and a single b-value of 1000 s/mm^2^ which is not optimal for CSD or apparent fiber density modelling. Indeed, it has been shown that the best conditions to perform CSD were higher b values and higher angular resolution^64^ and that both parameters influence apparent fiber density measure^65^. Thus, our measure of apparent fiber density might not be optimal and might influence our finding of wide overlap between FD and FA changes. While changes in FD might partly underlie changes in FA, the link between these two measures might also be due to the lower diffusion weighting limiting the access to the intra-axonal signal. By generating the tractogram with 5 million fiber tracts with a probabilistic algorithm, we try to reduce the influence of the relatively low b-value. Furthermore, to overcome the potential limitations of our comparably small number of diffusion directions or shells, we optimized each subject's final tractogram by the COMMIT framework. Although our diffusion-weighted dataset featured only one shell, it did not impair the COMMIT optimization with regard to the intracellular compartment fraction (referred to as FD) as demonstrated by Sommer and colleagues^66^ and in the appendix of Stämpfli and colleagues^46^.

Fourthly, although the COMMIT-based approach has not yet been evaluated by histological methods to confirm its microscopic validity, a correspondence between fiber weights -used to calculate FD—and myelin map has been shown thus suggesting a true biological meaning for FD^66^.

Finally, despite many advantages, TBSS has several limitations as reported by Bach and colleagues^67^. Prominently, the skeletonization steps restricts the analysis to regions with FA above a certain value (in this study we chose a typical value of 0.2). Thus, FA differences may be missed in regions with lower FA such as the perimeter of the tracts.

Conclusively, our results demonstrate that apparent fiber density is lower in premature-born adults. The more premature/or with lower birth weight an adult was born the lower its apparent fiber density which was also found to be correlated with lower full-scale IQ. Our data suggest that lower apparent fiber density at least partly underpins FA reductions of premature birth but that other processes such as hypomyelination need to be considered in future studies.

## Material and methods

### Participants

Participants were recruited from the Bavarian Longitudinal Study^68,69^. The BLS investigates a geographically defined whole-population sample of neonatal at-risk children and healthy term controls. All live-births born between January 1985 and March 1986 in Southern Bavaria and requiring admission to neonatal units in 17 children’s hospitals within the first ten days of life, comprised the target sample^69^. These included 682 born very preterm (VP; < 32 weeks of gestation) and/or with very low birth weight (VLBW; < 1500 g). During the same period, 916 healthy full-term infants (> 37 weeks of gestation; normal postnatal care) born in the same hospitals were recruited as control infants and 350 followed up beyond 5 years of age. At the age of 26 years there were 411 VP/VLBW survivors still living in Germany and 308 full term-born (FT) individuals. Two hundred and sixty VP/VLBW (63.2%) and 229 (74.4%) FT participated in psychological assessments. For details on the sample see Eryigit-Madzwamuse et al.^70^. FT individuals were selected to match the VP/VLBW group regarding the overall distribution of sex, family socio-economic status (SES) and maternal age. All 260 subjects from the VP/VLBW group who participated in the follow-up assessment at the age of 26 years underwent an initial screening for MR-related exclusion criteria, which included: (self-reported) claustrophobia, inability to lie still for > 30 min, unstable medical conditions (e.g. severe asthma), epilepsy, tinnitus, pregnancy, non-removable, MRI-incompatible metal implants and a history of severe central nervous system trauma or disease that would impair further analysis of the data. However, the most frequent reason not to perform the MRI exam was a lack of motivation. Characteristics of an interim subsample of this cohort has been described in^2^. The remaining eligible, 101 VP/VLBW and 111 FT individuals underwent MRI at 26 years of age. The final cohort consisted of 73 VP/VLBW adults and 89 FT individuals (after exclusion based on several criteria described below). Among the 73 VP/VLBW individuals, 15 subjects had a GA = or > 32 weeks and a BW below 1500 g, 16 subjects had a GA < 32 weeks and a BW = or > 1500 g and 42 subjects had a GA < 32 weeks and a BW below 1500 g.

MRI assessments were carried out at two different sites, the Department of Neuroradiology at the Klinikum rechts der Isar from the Technische Universität München, (n = 145) and the Department of Radiology at the University Hospital of Bonn (n = 67). The study was carried out in accordance with the Declaration of Helsinki and approved by the local ethics committee of the Klinikum rechts der Isar and the University Hospital Bonn. Informed consent was obtained from all participants who received travel expenses and a payment for attendance.

### Birth-related variables

GA was estimated from maternal reports of the last menstrual period and serial ultrasounds during pregnancy. When the two measures differed by more than two weeks, clinical assessment with the Dubowitz method was applied^71^. BW and maternal age were obtained from obstetric records. Neonatal medical complications were assessed with a standardized optimality scoring system (OPTI, neonatal optimality;^69–72^) which included 21 items (e.g. ventilation or intubation, sepsis, neonatal seizures, cerebral haemorrhage). Each item was coded as 1 (non-optimal) or 0 (optimal) with the higher value being less optimal and summarized.

### Cognitive assessment

Cognitive assessment at the age of 26 years was carried out by independently trained psychologists blinded to group membership. An abbreviated German version of the Wechsler Adult Intelligence Scale-III (WAIS III;^73^) was used to estimate FS-IQ performance.

### MRI data acquisition

The MRI data acquisition protocol used in this study was previously described in^2^:

Both whole-brain T1 and diffusion-weighted imaging data were acquired in Munich and Bonn, either on a 3 T Philips Achieva [Philips Medical System, Netherlands] or 3 T Philips Ingenia [Philips Medical System, Netherlands] MRI scanner using standard 8-channel SENSE head-coils. Sequence parameters were identical across scanners. To account for possible confounds by the scanner-specific differences, scanner identities were included in the analyses as covariates of no interest. The distribution of subjects among scanners was as follows: Bonn Achieva 3 T: 5 VP/VLBW, 12 FT, Bonn Ingenia 3 T: 33 VP/VLBW, 17 FT, Munich Achieva 3 T: 60 VP/VLBW, 65 FT, Munich Ingenia 3 T: 3 VP/VLBW, 17 FT.

Diffusion-weighted imaging was performed by single‐shot spin‐echo echo‐planar imaging sequence, resulting in one non‐diffusion weighted image (b = 0 s/mm^2^) and 32 diffusion weighted images (b = 1,000 s/mm^2^, 32 non-collinear gradient directions) covering whole brain with the following parameters: SENSE factor = 2, echo time (TE) = 47 ms, repetition time (TR) = 20,150 ms, flip angle = 90°, field of view = 224 × 224 mm^2^, matrix = 112 × 112, 75 transverse slices, slice thickness = 2 mm, 0 mm interslice gap and voxel size = 2 × 2 × 2 mm^3^.

A whole head high resolution T1-weighted dataset was acquired using a magnetization-prepared rapid acquisition gradient echo (MPRAGE) sequence with the following parameters: TI = 1300 ms, TR = 7.7 ms, TE = 3.9 ms, flip angle 15°; field of view: 256 mm × 256 mm, 180 sagittal slices, slice thickness = 1 mm, 0 mm interslice gap and a reconstructed isotropic voxel size of 1 mm^3^.

### Diffusion weighted data preprocessing and quality check

Both preprocessing and quality check of diffusion data followed a procedure similar to the one previously described in^47^. In short, the diffusion weighted data were first denoised using the "dwidenoise function" from the MRtrix3 software package (https://www.mrtrix.org) and subsequently corrected for eddy-current and motion induced distortions by registration of the diffusion weighted images to the b0 image using the dwipreproc routine from MRtrix3. This function makes use of the eddy tool implemented in FSL (^74^; FMRIB, Oxford, UK version 6.0.0). The brain extraction tool (BET) from FSL was then applied to remove non-brain tissue and estimate the inner and outer skull surfaces. As a next step, the quality of all diffusion datasets was assessed based on the following criteria: (1) tensor residuals that were calculated for each diffusion direction and visually inspected for the nine slices with highest residuals in the whole diffusion dataset, (2) mean intensity plots that were derived slice by slice in sagittal, axial and coronal direction for each diffusion direction and non-diffusion-weighted images. Head motion normally induces peaks which can easily be spotted on these plots. Based on these measures and additional visual inspection of DWI data, each dataset was classified has having no, slight or strong artifacts. Only participants with no artifacts were kept, thus leading to the exclusion of 19 participants due to motion artifacts, 16 because of insufficient fat suppression (ghosting) artifacts and 1 due to corrupted data. From the original cohort of 210 participants with available diffusion datasets, 12 participants were additionally excluded for having a sibling already taking part in the study.

### Diffusion data analysis and parameters calculation

Prior to the calculation of FD and FA maps, the diffusion data were corrected for susceptibility-induced distortions using the bdp correction algorithm implemented in the BrainSuite software package (https://brainsuite.org) (for more details see the method described in^75^). In brief, the information from each subject's MPRAGE image is used to estimate a deformation map and un-distort the diffusion data via constrained non-rigid registration. Subsequently, single shell constrained spherical deconvolution with recursive calibration of the response function^45,76^ and fiber tractography was performed using MRtrix^60^. For tractography, the iFOD2 probabilistic tractography algorithm was used with the "Anatomically Constrained Tractography" (ACT) option (MRtrix3 tckgen act option) in order to apply biological tissue priors to the streamline generation^59^. Seed points for tractography were determined dynamically according to the spherical-deconvolution informed filtering of the tractogram (SIFT) model (MRtrix3 tckgen seed-dynamic option for determining seed points based on the SIFT model from^77^). As this dynamic seeding strategy takes place within the whole WM, the distribution of streamlines is already approximating the fiber density and thus reducing intrinsic tractography biases. In total, five million fibers were generated for every tractogram. The resulting streamlines were optimized using the COMMIT framework^42^ and the parameters previously described in^78^ to get a weight for every reconstructed fiber. By modelling and optimizing the contribution of each compartment accordingly and summing up the fiber weights in each voxel, FD maps—which correspond to the resulting intracellular compartment fraction—were derived. Finally, FA maps were calculated using the DTIFIT tool implemented in the FSL software package. An example of FA and FD maps can be found in supplementary material (Supplementary Fig. S1).

### Skeletonized FA generation and tract based spatial statistics

Voxel-wise statistical analyses of FD and FA data were carried out using Tract Based Spatial Statistics^79^. All subjects FA images were first non-linearly registered and aligned to the Montreal Neurological Institute Standard Space (MNI 152.2 mm standard space). Next, the mean FA image of all subjects was created and used to generate an across-all-subjects skeleton, which represent the WM tracts common to all subjects. We then thresholded the skeleton to FA > 0.2 to keep the main WM tracts only before projecting each subject's FA image onto the skeleton to obtain individual FA maps.

Finally, FD maps were analyzed using the tbss_non_FA script which applies the same non-linear registration, warping and skeleton projection operations as for the corresponding FA maps.

### Statistical analysis

Differences of clinical variables between VP/VLBW and FT individuals were tested using Chi^2^ tests (sex, SES), two-sample t-tests (age, FS-IQ) and Mann–Whitney U tests (GA, BW, OPTI, maternal age).

### Whole brain group differences

Voxelwise group comparisons in FA and FD were performed based on the General Linear Model (GLM) and using the FSL randomize tool with 5000 permutations. Sex and scanner type were added as covariates of no interest in the model and the statistical threshold was set at p < 0.05 after threshold-free cluster enhancement (TFCE) and family-wise error correction (p_FWE_ < 0.05, TFCE corrected). Regions of statistical significance were identified using the John Hopkins University (JHU) -ICBM-DTI-81 WM labels atlas^80^. Each structure was used as a mask and binarized to extract the number of significant voxels for each label. Significant voxels that did not belong to a structure were referred to as unclassified voxels.

Significantly lower FD and FA voxels were binarized to create masks of significant lower FD and lower FA respectively in VP/VLBW individuals. By multiplying them, a mask of the overlap between the map of lower FD and the map of lower FA (Fig. 4) was created and the number of voxels it contained was calculated.

### Correlation with birth related variables and FS-IQ

Across VP/VLBW adults, linear relationships between FD values and several variables of interest were investigated using a GLM in the clusters of WM voxels that showed significant group differences. First, in order to link lower FD to the degree of prematurity, voxelwise correlation analyses were performed between FD and birth-related variables as indexed by GA, BW or OPTI score. Second, in order to investigate the relevance of lower FD for FS-IQ, a voxelwise correlation analysis was performed between FD and FS-IQ. As for the group comparison approach, 5000 permutations were used and sex and scanner were added as covariates of no-interest. Statistical significance was set at p < 0.05 after threshold-free cluster enhancement (TFCE) and family-wise error correction (pFWE < 0.05, TFCE corrected).

For better visualization, all significant clusters were displayed using the tbss_fill script which thickens the results filling them out into the local tracts seen in the mean FA image.

## Supplementary information

Supplementary Information.

## Data Availability

The data supporting the findings of this study are available on request from the corresponding author. The data used in this study are not publicly available due to restrictions by the Bavarian Longitudinal Study.

## Abbreviations

ACT: Anatomically constrained tractography
BET: Brain extraction tool
BLS: Bavarian longitudinal study
BW: Birth weight
COMMIT: Convex optimization modeling for microstructure informed tractography
CSD: Constrained spherical deconvolution
CSF: Cerebrospinal fluid
DTI: Diffusion tensor imaging
DWI: Diffusion weighted imaging
FA: Fractional anisotropy
FD: Fiber density
FS-IQ: Full-scale intelligence quotient
FT: Full-term
FWE: Family-wise error
GA: Gestational age
GLM: General linear model
IQ: Intelligence quotient
MNI: Montreal neurological institute
MPRAGE: Magnetization prepared rapid acquisition gradient echo
MRI: Magnetic resonance imaging
OPC: Oligodendrocyte progenitor cell
OPTI: Standardized optimality scoring system
pre-OL: Pre-oligodendrocytes
SES: Socioeconomic status
SIFT: Spherical deconvolution informed filtering of the tractogram
TBSS: Tract-based spatial statistics
TE: Echo time
TFCE: Threshold-free cluster enhancement
TR: Repetition time
VP/VLBW: Very preterm and/or very low birthweight
WM: White matter
