# Supplementary material for: Reduced apparent fiber density in the white matter of premature-born adults
Source: Sci Rep. 2020 Oct 14;10:17214. doi: 10.1038/s41598-020-73717-6 (PMC7560721; doi:10.1038/s41598-020-73717-6)

**Title:**

**Reduced apparent fiber density in the white matter of premature-born adults**

**Authors and Affiliations:**

Aurore Menegaux^1,2^, Dennis M. Hedderich^1,2^, Josef G. Bäuml^1,2^, Andrei Manoliu^3,4,5^, Marcel Daamen^6,7^, Ronja C. Berg^1,2^, Christine Preibisch^1,2^, Claus Zimmer^1,2^, Henning Boecker^6^, Peter Bartmann^7^, Dieter Wolke^8,9^, Christian Sorg^1,2,10^, Philipp Stämpfli^3,11^

^1^Department of Neuroradiology, School of Medicine, Technical University of Munich, Munich, Germany ^2^TUM Neuroimaging Center, School of Medicine, Technical University of Munich, Munich, Germany; ^3^Department of Psychiatry, Psychotherapy and Psychosomatics, Psychiatric Hospital, University of Zurich, Zurich, Switzerland; ^4^Wellcome Centre for Human Neuroimaging, University College London, London, United Kingdom; ^5^Max Planck University College London, Centre for Computational Psychiatry and Ageing Research, London, United Kingdom; ^6^Functional Neuroimaging Group, Department of Radiology, University Hospital Bonn, Bonn, Germany; ^7^Department of Neonatology, University Hospital Bonn, Bonn, Germany; ^8^Department of Psychology, University of Warwick, Coventry, UK; ^9^Warwick Medical School, University of Warwick, Coventry, UK; ^10^Department of Psychiatry, School of Medicine, Technical University of Munich, Munich, Germany; ^11^MR-Center of the Psychiatric Hospital and the Department of Child and Adolescent Psychiatry, University of Zurich, Zurich, Switzerland

**Supplementary material**

***Table S1: Number of voxels and atlas localization of group differences in FD and FA***

| **JHU ROI** | **FD in VP/VLBW < FT cluster size (voxels)** | **FA in VP/VLBW < FT cluster size (voxels)** | **Overlap lower FD-FA cluster size (voxels)** |
| --- | --- | --- | --- |
| Middle cerebellar peduncle | 0 | 169 | 0 |
| Pontine crossing tract | 0 | 113 | 0 |
| Genu CC | 1460 | 853 | 804 |
| Body CC | 2766 | 2414 | 2151 |
| Splenium CC | 1585 | 761 | 691 |
| Fornix (column + body) | 126 | 129 | 126 |
| CST R | 0 | 2 | 0 |
| CST L | 0 | 3 | 0 |
| Medial lemniscus R | 2 | 71 | 1 |
| Medial lemniscus L | 0 | 31 | 0 |
| Inferior cerebellar peduncle R | 0 | 161 | 0 |
| Inferior cerebellar peduncle L | 0 | 113 | 0 |
| Superior cerebellar peduncle R | 146 | 183 | 126 |
| Superior cerebellar peduncle L | 0 | 159 | 0 |
| Cerebral peduncle R | 413 | 450 | 362 |
| Cerebral peduncle L | 22 | 207 | 17 |
| ALIC R | 134 | 204 | 111 |
| ALIC L | 0 | 55 | 0 |
| PLIC R | 586 | 537 | 428 |
| PLIC L | 180 | 227 | 68 |
| RLIC R | 232 | 153 | 116 |
| RLIC L | 249 | 477 | 229 |
| ACR R | 222 | 319 | 207 |
| ACR L | 230 | 389 | 173 |
| SCR R | 455 | 363 | 294 |
| SCR L | 196 | 225 | 109 |
| PCR R | 176 | 168 | 105 |
| PCR L | 450 | 332 | 302 |
| PTR (including optic radiations) R | 362 | 704 | 349 |
| PTR (including optic radiations L | 372 | 690 | 354 |
| Sagittal Stratum R (including ILF + IFOF) | 186 | 158 | 129 |
| Sagittal Stratum L (including ILF + IFOF) | 158 | 146 | 108 |
| EC R | 639 | 942 | 558 |
| EC L | 345 | 1077 | 288 |
| Cingulum (gyrus) R | 221 | 287 | 220 |
| Cingulum (gyrus) L | 34 | 319 | 34 |
| Cingulum (hippocampus) R | 107 | 161 | 51 |
| Cingulum (hippocampus) L | 8 | 78 | 4 |
| Fornix cres / striaT R | 181 | 178 | 155 |
| Fornix cres / striaT L | 251 | 270 | 238 |
| SLF R | 256 | 242 | 52 |
| SLF L | 0 | 352 | 0 |
| SFOF R | 22 | 47 | 21 |
| SFOF L | 0 | 4 | 0 |
| Uncinate fasciculus R | 56 | 65 | 51 |
| Uncinate fasciculus L | 25 | 60 | 25 |
| Tapetum R | 16 | 20 | 16 |
| Tapetum L | 2 | 2 | 2 |
| Non classified voxels | 18027 | 35930 | 9075 |

**Abbreviations:** R, right; L, left; CC, corpus callosum; CST, corticospinal tract; ALIC, anterior limb of internal capsule; PLIC, posterior limb of internal capsule; RLIC; retrolenticular part of internal capsule; ACR; anterior corona radiata; SCR, superior corona radiata; PCR:, posterior corona radiata; PTR, posterior thalamic radiation; ILF, Inferior longitudinal fasciculus; IFOF, inferior fronto-occipital fasciculus; EC, external capsule; StriaT, stria terminalis; SLF, superior longitudinal fasciculus; SFOF, superior fronto-occipital fasciculus;

***Table S2: Number of voxels and atlas localization of the association between FD and prematurity variables in VP/VLBW adults***

| **JHU ROI** | **Association between FD and GA in VP/VLBW** | **Association between FD and GA in VP/VLBW** |
| --- | --- | --- |
| Anterior Corona Radiata R | 99 | 40 |
| Anterior Corona Radiata L | 63 | 0 |
| Superior Corona Radiata R | 2 | 7 |
| Superior Corona Radiata L | 7 | 0 |
| Genu of the Corpus Callosum | 695 | 51 |
| Body of the Corpus Callosum | 509 | 496 |
| Fornix | 91 | 0 |
| Cerebral Peduncle R | 178 | 0 |
| Posterior limb of the internal capsule R | 72 | 0 |
| Retrolenticular part of the internal capsule R | 89 | 0 |
| Retrolenticular part of the internal capsule L | 1 | 0 |
| Sagittal Stratum R | 20 | 0 |
| External Capsule R | 84 | 0 |
| Fornix cres Stria Terminalis R | 122 | 0 |
| Fornix cres Stria Terminalis L | 59 | 0 |
| Uncinate R | 3 | 0 |
| Non classified voxels | 1365 | 106 |

**Abbreviations:** R, right; L, left

***Table S3: Number of voxels and atlas localization of the association between FD and FS-IQ in VP/VLBW adults***

| **JHU ROI** | **Association between FD and IQ in VP/VLBW** |
| --- | --- |
| Anterior Corona Radiata R | 107 |
| Superior Corona Radiata R | 102 |
| Superior Corona Radiata L | 5 |
| Genu of the Corpus Callosum | 735 |
| Body of the Corpus Callosum | 1603 |
| Splenium of the Corpus Callosum | 152 |
| Non classified voxels | 61 |

**Abbreviations:** R, right; L, left

***Supplementary Figure S1: Example of a FA and a FD map for one subject***

Axial views of a FA (left) and a FD map (right) presented, using FSLView Version 5.0.9 (<https://fsl.fmrib.ox.ac.uk/fsl/fslwiki/FslView>).


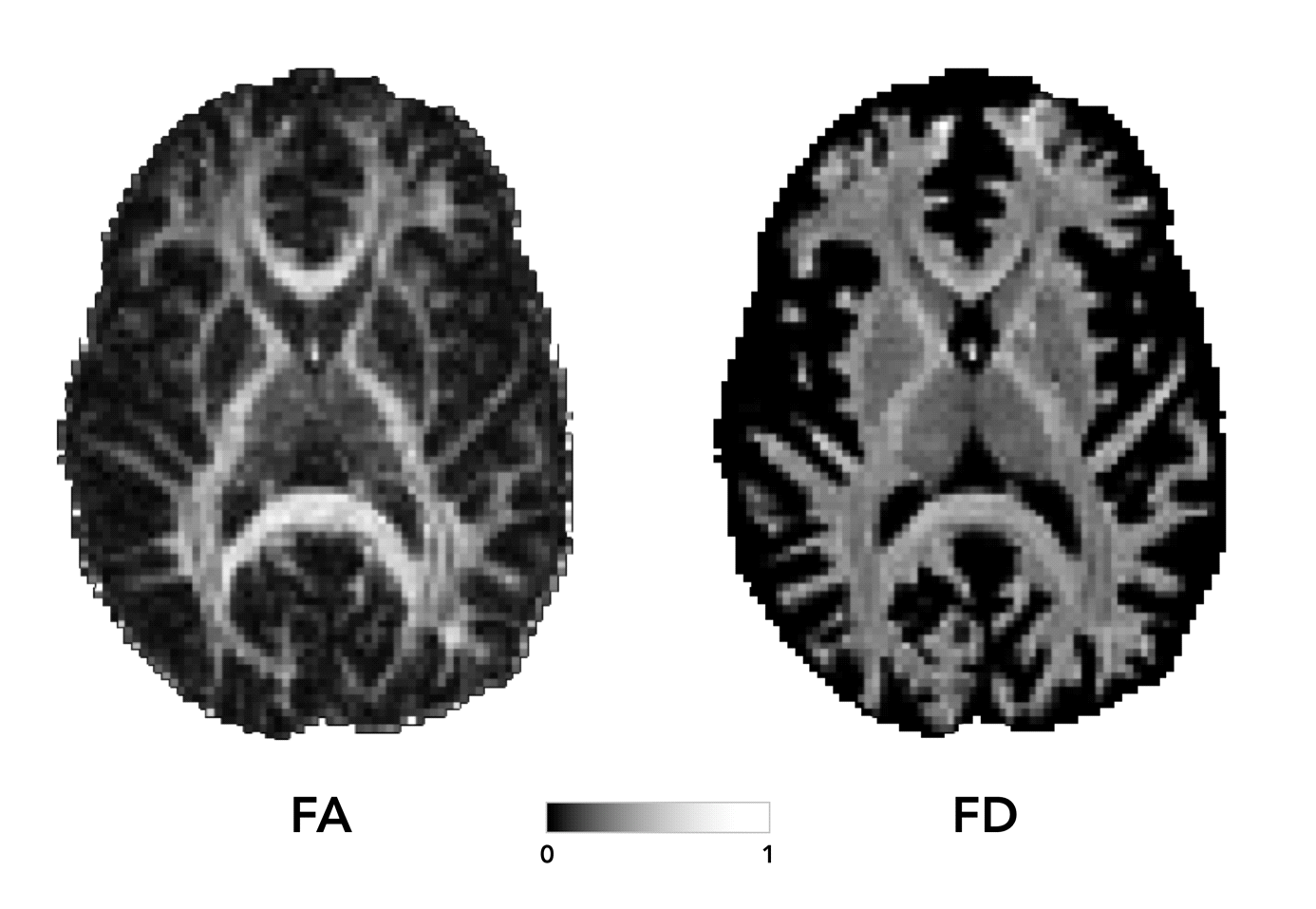


***Supplementary Figure S2: Lower FA in VP/VLBW individuals***

**A.** Coronal, axial, and sagittal views illustrating the group differences in FA between VP/VLBW adults and FT individuals overlaid on the T1-weighted brain image of MNI152 structural standard template using FSLView Version 5.0.9 (<https://fsl.fmrib.ox.ac.uk/fsl/fslwiki/FslView>). Clusters of voxels significantly reduced in VP/VLBW adults (p<.05) are represented in red on the green TBSS FA skeleton. MNI coordinates are provided at the bottom.


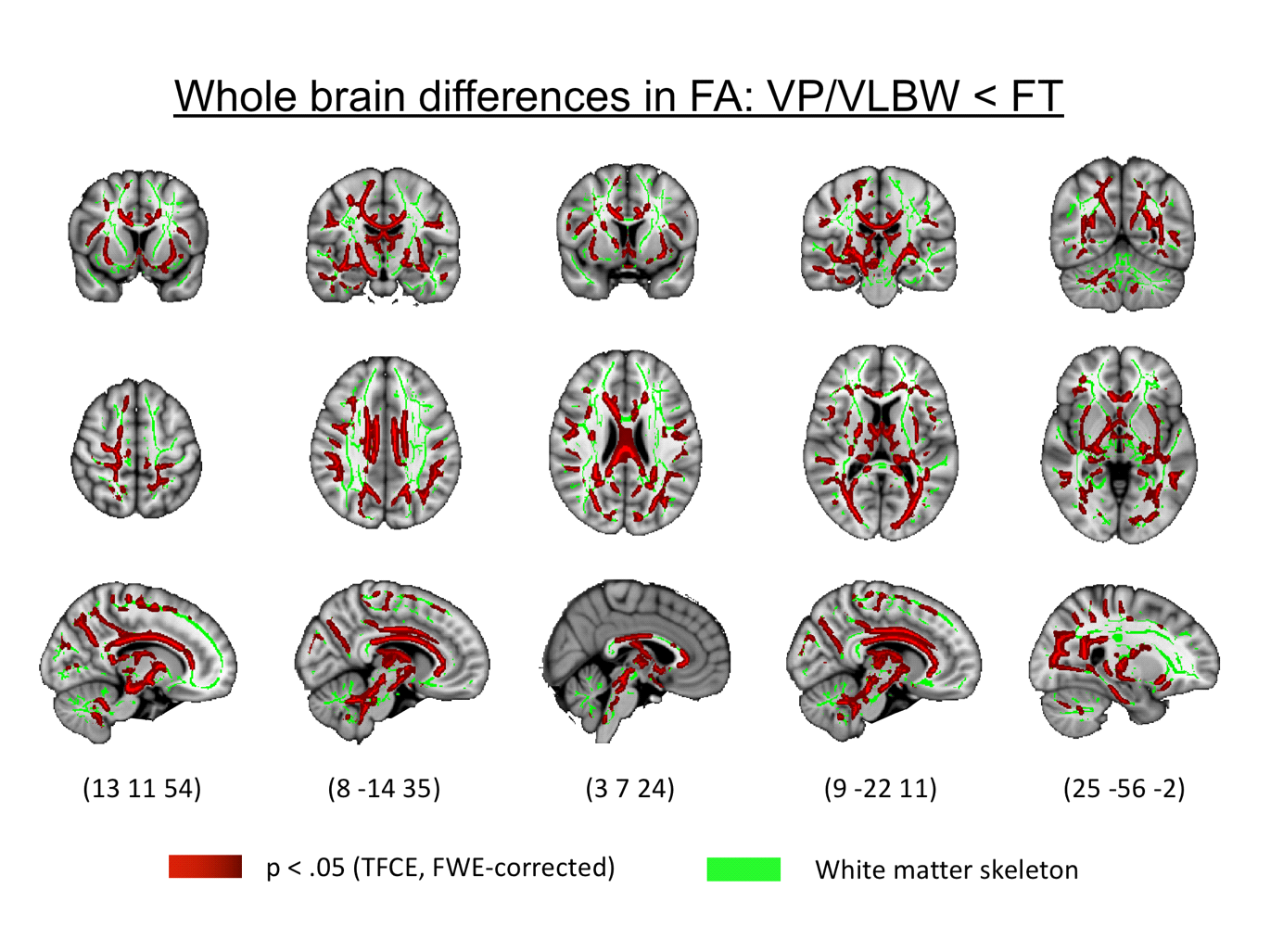

Supplement: Supplementary file 1 — Supplementary Information. [file 41598_2020_73717_MOESM1_ESM.docx]
